# Reasons for participation and experiences of women in an antibody-mediated HIV prevention clinical trial in Malawi

**DOI:** 10.11604/pamj.2026.54.16.52074

**Published:** 2026-05-19

**Authors:** Ruth Naomi Holla, Agatha Bula, Adamson Muula, Mina Hosseinipour

**Affiliations:** 1 World Health Organization, Lilongwe, Malawi,; 2 UNC Project, Lilongwe, Malawi,; 3 Kamuzu University of Health Sciences, Chichiri, Blantyre 3, Malawi

**Keywords:** HIV, antibody-mediated prevention study, clinical trial, informed consent, Malawi

## Abstract

**Introduction:**

in the Antibody-Mediated Prevention (AMP) trials, a broadly neutralizing antibody called VRC01 was infused intravenously every two months to test its safety and HIV prevention efficacy. The AMP trial conducted in sub-Saharan Africa, called HVTN 703, enrolled women without HIV who were not pregnant or breastfeeding but who were behaviorally vulnerable to HIV-1 infection. We conducted a qualitative study in Malawi to explore the motivations and experiences of trial participants.

**Methods:**

between September and December 2018, we purposively selected and interviewed 12 out of 100 women enrolled in the AMP trial at the UNC Project site in Lilongwe, Malawi. We conducted in-depth interviews (IDI) with women who were retained in the trial. All IDIs were conducted in Chichewa, digitally recorded, and then transcribed into English. Data was analyzed using thematic analysis.

**Results:**

women participated in the AMP trial due to a range of perceived benefits. The primary reason was access to better quality medical care, accompanied by financial benefits and altruism. Participants' research experiences and their continued participation in the AMP trial were influenced by the research clinic environment and the nature of their interactions with research staff. Regardless of pre-existing study expectations, feeling welcomed and being able to openly interact with the study staff encouraged them to continue participating in the study.

**Conclusion:**

antibody-mediated prevention study participants enrolled and continued their participation because of perceived benefits such as access to quality health care, finances, and altruism. Provision of other health services to research participants promotes participation in the trials.

## Introduction

Malawi continues to make progress in the fight against HIV and AIDS. HIV prevalence has decreased from 10.6% to 8.8% among women and men aged 15-49 in the years, with a corresponding decrease in HIV infection rate by 46% between 2010 and 2015-16 [1], UNAIDS, 90 90 90. However, despite the progress, HIV prevalence among young women is twice as high as that of their male counterparts within the same age range, and over 37,000 new infections are estimated to occur each year in Malawi, with women under 30 bearing the highest proportion of new infections [2].

Several HIV prevention strategies have proved to be highly effective in reducing the likelihood of acquiring HIV. These methods include the use of internal and external condoms, antiretroviral medicines used as both post-exposure prophylaxis (PEP) and pre-exposure prophylaxis (PrEP), voluntary male medical circumcision (VMMC), behavior change interventions aimed at reducing the number of sexual partners, the use of clean needles and syringes, and the treatment of people living with HIV to reduce viral load and prevent onward transmission [3]. Additionally, female-initiated HIV prevention technologies remain critical, as many women are unable to negotiate the current HIV prevention options without partner support [4].

Clinical trials are a crucial step in providing scientific evidence of diagnostic and therapeutic safety and efficacy in humans [5]. Participants´ involvement and retention are crucial in HIV clinical trials, hence, improved understanding of research participants´ experiences with respect to autonomy, safety, and satisfaction can help researchers enhance human subjects´ protection, including informed consent; enhance recruitment and retention; improve the quality of clinical research processes; and increase public trust in the research enterprise [6]. HIV Prevention Trials Network (HPTN) studies evaluate new HIV prevention interventions and strategies in populations and geographical regions that bear a disproportionate burden of infection. This is intended to facilitate rapid scale-up of proven interventions and to have the greatest possible impact on the pandemic [7]. One of the clinical trials is the AMP study, co-sponsored by the HIV vaccine trials network (HVTN) and the HPTN [8]. The AMP study found VRC01 was not effective in preventing HIV acquisition unless the individual´s HIV strain was fully sensitive to VCR01 [8]. The study enrolled healthy, non-pregnant, non-breastfeeding women without HIV in sub-Saharan Africa, including Malawi, who are behaviorally vulnerable to HIV-1 infection [9,10].

The perceptions and experiences of women taking part in HIV prevention trials were explored in Soweto, South Africa, whereby the central finding was that the participants felt a sense of empowerment in spite of their being embedded in a culture that has come to fear, deny, or ignore AIDS [11]. In similar studies conducted in Malawi, the majority participated in research for the sake of obtaining better quality treatment made available through the clinical trials as ancillary care, and the monetary and material incentives given to them [12,13]. Currently, there is limited information on the reasons and experiences of research participants in the AMP study. The aim of this study was to assess the reasons, experiences, and challenges of female participants in the AMP study, Lilongwe research site.

## Methods

**Study setting:** the study was conducted in Lilongwe, the capital city of Malawi, with an estimated population of 1,753,299. The city has an HIV prevalence of approximately 10.6% and an HIV incidence rate of 0.07%, which is among the highest in the Central Region of Malawi. Among the adult population, unemployment is estimated at 16%, and food insecurity affects approximately 11% of households.

**Study design and population:** we conducted an exploratory qualitative study between September 2018 and December 2018 at the UNC Project located within the Kamuzu Central Hospital (KCH) Campus, Lilongwe, Malawi. Lilongwe is the capital of Malawi, the largest city in Malawi, with a population of 1,753,299. HIV prevalence is estimated at 10.6%, and the HIV incidence is 0.07%, the highest in the central region of Malawi. Among the adult population, unemployment is 16%, and food insecurity is prevalent (11%). Exploratory qualitative studies are used to gain an understanding of underlying reasons, opinions, and motivations; hence, we used qualitative research methods to gain an in-depth understanding of participants´ reasons and experiences in participating in HIV prevention clinical trials, mainly the AMP research study.

The study population included women already enrolled in the ongoing AMP study, one of the HIV prevention clinical trials being conducted on site [9]. We purposively recruited and interviewed 12 women among the 100 participants who were enrolled and actively participating in the AMP study. Participants were recruited during their monthly face-to-face study follow-up visits. Participants were eligible if they met the following criteria: enrolled in the AMP research study, continuing with their study visits, aged between 18 and 35 years, and willing to provide informed consent. The research nurses from the main AMP study briefly explained the qualitative study to the study participants, and those interested were referred to the research assistant. The research assistant then explained the purpose of this qualitative study, and written informed consent was obtained from those willing to participate. Recruitment continued until there was data saturation and redundancy.

**Data collection:** IDIs were used to explore views, feelings, and perspectives of study participants in relation to their participation in the AMP study. This allowed the participants to express their story in their own voice. All the interviews were done by a female qualitative research associate working at the UNC Qualitative Unit, who was not directly involved in the AMP study and was recruited based on her vast experience in conducting qualitative research. She was trained for one day on the study protocol, recruitment procedures, and data collection tools. The IDIs were conducted on the same days as AMP trial clinic visits at the UNC Project, Lilongwe, but away from the clinic rooms where the trial visits were conducted. The IDIs were conducted using an interview guide that outlined major topics that were aimed at exploring the participants´ experiences of participating in the HIV prevention clinical trials and the challenges faced. The interviews lasted for a maximum of 30 minutes and were audio recorded with participants´ consent to ensure that all vital information was captured. The lead researcher conducted spot checks during data collection to ensure compliance with the study protocol. Field notes were also recorded throughout the data collection period to capture context that could not be captured through recording. At the end of each day, the lead researcher met with the research assistant to discuss emerging themes and address any challenges faced during data collection.

**Data management and analysis:** audio recordings were transcribed and translated from Chichewa to English by the research assistant. Each transcription was labelled with the interview identification code, which was assigned to the participant during the interview. Transcripts were then proofread by the lead researcher to get familiar with the content and ensure consistency of data. Emergent themes and codes were extracted from data review, and these were used to develop a codebook, which helped with the analysis. Similar themes were categorized accordingly, and data were analyzed manually using thematic analysis. Thematic analysis is the process of identifying patterns or themes within qualitative data, which involves a rigorous process of data familiarization, data coding, theme development, and theme revision [14]. The coded data were then used to construct matrices to summarize the data.

**Ethical considerations:** the study received ethical clearance from the College of Medicine Research and Ethics Committee (COMREC) (Ref No.: P.08/18/2466). Further permission was obtained from the UNC Project management for the study to be conducted in the respective HIV prevention clinical trial. To ensure study participants´ privacy, all study staff were trained in human subjects´ protection and good clinical practices. Therefore, the participants´ information was treated with privacy and confidentiality. All participants provided written informed consent prior to participation, and all study participants were informed of all potential risks related to their participation in the study. Participants were also informed of their right to withdraw from the study and not to answer any questions they did not feel comfortable answering.

## Results

**Characteristics of participants:** fourteen study participants were informed of the sub-study, of which twelve consented to participate. All twelve AMP study participants were HIV negative. Their ages ranged from 18 to 35, and the majority had attended some primary school education and were unemployed. The other two refused participation due to time constraints. Table 1 summarizes the participants´ demographic characteristics.

**Table 1:** socio-demographic characteristics of women participating in the antibody-mediated prevention HIV prevention clinical trial at UNC Project, Lilongwe, Malawi, September-December 2018 (N=12)

Characteristic	Frequency
**Age**	
16-20	1
21-25	4
26-30	5
31-35	2
**Education**	
None	1
Primary	8
Secondary	3
**Employment**	
Employed	4
Unemployed	8

**Level of knowledge on the AMP study:** nine of the 12 interviewed participants were highly knowledgeable about the AMP study and managed to explain what the research means to them and the aim of the research study. They were able to explain the main objective of the AMP study and what happens during their study visits. *“In the AMP study, they want to test a certain type of drug in our body and also to know if the drug can fight HIV or not”* (respondent #8). *“They insert drips on us, but it´s not like when they put it, they tell us to go and have unprotected sex, no. They are encouraging us to use protection; they are giving us condoms because they don´t really know if the drips we are receiving are just a placebo or if we have received the active drips, the ones with VRC01. So, they are just trying it out”* (respondent #3).

A few participants were less knowledgeable about the AMP study, as their explanations were less complete or confident. When asked to explain their knowledge about the study, one study participant had this to say: *“The AMP study, I know that (silence) like, how to prevent…how we can prevent the diseases, they give us umm, they give us the drips so that if someone has HIV, they should not transmit it to us. Like, we have also read some books before on ways of preventing yourself from contracting diseases using this medicine, and things like that”* (respondent number 4).

Participants mentioned two main sources where they got the information about the AMP study: their friends and the UNC Project community educators who conduct sensitization meetings in the community as part of the recruitment and retention strategy of study participants. Previous and current prevention trial participants at the UNC Project frequently provided information to potential participants. During the interviews, some respondents narrated: *“A friend of mine told me that there is also a study being conducted at Central (Meaning Kamuzu Central Hospital). From what my friend was telling me, she said that the study was very hard, but I just said okay, I will try it out”* (respondent #6).

The second source of information mentioned was through the community educators from the UNC Project, who conduct sensitization meetings in the community as part of the recruitment and retention strategy of study participants. One of the respondents who got the AMP study information from the UNC community educator had to say: *“For me to know of the AMP study, we were in a learning group. While in this group, we would meet with doctors who provide services to females who are promiscuous (prostitutes), so they chose us to be part of that group, and we would learn there. Whilst we were learning, a doctor came, and he said that “we are not forcing anyone, but for those who can volunteer themselves, those who can manage, there is a study being conducted at the Central Hospital, Tidziwe Centre. Those who want can enroll, and those who do not want will not enroll. We volunteered ourselves, from our group, 2 of us volunteered ourselves, me and a certain friend of mine…”* (respondent #3).

**Motivating factors to enroll in the study:** participants were asked about the reasons for participating in the AMP study. Many participants reported that they were motivated to join the AMP study because they anticipated receiving health benefits, mainly because of the laboratory tests that identify health problems they have and the treatment and counselling received during each study visit. *“When they take the blood samples, they test for different diseases, and they find diseases which you did not even think you would have; gonorrhea, syphilis, even AIDS. So that is what I liked… when we are sick, they give us enough medications”* (respondent #2). *“The benefits I have seen are that…it is good because it encourages me to protect myself against HIV. Thinking of it, it is really good to protect myself. So I think that to me, it is good”* (respondent number 4).

Another motivating factor reported by other participants was their willingness to help the society and the Government of Malawi to reduce HIV transmission through their participation in the study. They believed that the study findings would help the country to find new ways to prevent further spread of HIV, especially among women. *“I just prepared myself to take part since I was told that the drug that I will be getting might benefit a lot of people if they work better in my body”* (respondent number 8). Some participants were motivated to join the study because they felt behaviorally vulnerable to HIV due to the nature of their work (sex work) or because they or their sexual partners had multiple sexual partners. *“You know the life of a girl nowadays, you cannot just have one boyfriend, that is impossible. So I just said okay, I will enroll in the study, considering how I am, I will enroll. Some of us get heartbroken a lot, and so I don´t have one boyfriend, so I said I would enroll in the study. So when I came and heard, I was satisfied to enroll”* (respondent #4).

On the other hand, some participants reported having been motivated to join the study because of financial benefits through transport reimbursement and gifts. Some of the interviewed participants said the following: *“I was told that you are given money and gifts which can make you enjoy”* (respondent #10). *“The good that I experienced in this study, I am not married, I just have casual partners, which is also why I enrolled in this study. My marriage ended a long time ago, in 2007. I have little children. One has gone to school, one started working, and the other one is in college. So, on the benefit of this study, the money you give me here, I am able to buy relish (food) food with that for 3 or 4 days, and the children eat”* (respondent #5). *“The benefit I have experienced, from the money I get here, I am able to buy clothes. Sometimes I am also able to share my mother”* (respondent number 7).

**Women's experiences during study participation:** all participants expressed that the study intervention, an infusion of VRC-01 antibody, is acceptable since they have not encountered any problems during their study participation. *“This method of drips has no problem. I should just say it is good, it does not have problems. We, people, have different bodies, but to me as an individual, I have no problem, I don´t have any problems with it”* (respondent number 6). The women also mentioned the friendliness, supportive, understanding, and accommodating nature of the research staff as one of the main factors that motivated them to participate and remain in the study. Feeling welcomed and being able to open up to the study staff were also one of the factors that encouraged their continued participation in the study. *“We are warmly welcomed, get the right assistance, and we get the right treatment when we are sick”* (respondent number 9). Additionally, they explained that they would recommend HIV prevention trials participation to their families and friends if given a chance. One of the respondents had the following to say: *“From what I have benefited from the study, I would encourage my friends and even my family members to join these kinds of studies. I would tell them the importance of the research studies; the rest would be up to them to decide”* (respondent number 12).

Additionally, some participants expressed a change in their lifestyle due to their participation in the HIV prevention AMP study. The following quote from an interview with one of the study participants was part of these responses: *“This study has still helped me because like I said, I had 5 boyfriends, and I would have unprotected sex with them, and I was not even getting tested… If I still led the same life I had before the study, would I be saying that I want to be an example? That would not have been possible because I don´t know what the others were doing, and it´s not like we were getting tested together, no”* (respondent number 3).

She further commented on the effect of the study on her sexual behavior: *“Since I enrolled in the study, I follow the counsel they give us here. They said that just because we are in the study, it does not mean that we should just have unprotected sex. They give us condoms, so we should use those to protect ourselves. So we listen to what they tell us. You cannot use condoms every day (laughs), there is still someone you can decide to just have sex without a condom with. That is very risky, however, because you don´t know their status, and you can only know if you have come and gotten tested together with them”*. The participants appreciated the more rapid service that they received at the study clinic compared to usual clinics or hospitals: *“When we arrive, they welcome us in time. If we are sick, they attend to us early and give us treatment on time so that we can go back home. This is different from other hospitals because we can go in the morning and come back in the evening”* (respondent #11).

**Challenges faced from participating in the study:** while the majority of the participants reported that they never experienced any challenge during their participation in the AMP study, some participants said that they were getting negative comments from their communities due to their participation in the study. *“Our friends were the ones saying a lot of things… they were saying that they are drawing our blood here and that when we are sick, we will have no one to share our blood”* (respondent number 2). *“In the communities, they say that they collect drips of blood from us and sell it to sick people, and yet they don´t collect drips of blood from us... There is no challenge which I have experienced here; it is only when we are home, and people are saying all those things. But there is no other challenge”* (respondent number 7).

**Challenges faced from participating in the study:** while the majority of the participants reported that they never experienced any challenge during their participation in the AMP study, some participants said that they were getting negative comments from their communities due to their participation in the study. *“Our friends were the ones saying a lot of things… they were saying that they are drawing our blood here and that when we are sick, we will have no one to share our blood”* (respondent number 2). *“In the communities, they say that they collect drips of blood from us and sell it to sick people, and yet they don´t collect drips of blood from us... There is no challenge which I have experienced here; it is only when we are home, and people are saying all those things. But there is no other challenge”* (respondent number 7).

## Discussion

This study found that the primary reason for the participants´ motivation to enroll and remain in the clinical trial was access to better quality medical care, accompanied by financial benefits and altruism. These findings agree to varying degrees with similar studies conducted in Malawi and Brazil that revealed access to quality care as a primary motivator to participate in clinical trials [15,16]. A systematic review found that 8 out of 12 studies reported that financial reward was the principal reason for participation in an HIV clinical trial [17]. In a similar study from Kenya, altruism served as a motivator, especially among volunteers who knew of someone infected with HIV [18]. Our study found all factors (access to care, financial benefit, and altruism) to be important motivators. This difference in the primary motivators could be related to perceived inadequacies of health care systems in the countries and individuals´ perceived susceptibility of contracting HIV among our study participants.

The National Institute for Health Research (NIHR) reports that the number of clinical trials and participants has increased progressively over the years. [18]. This change in participation may be attributed to the general awareness of the general communities about what research is and why to take part. While the study participants stated to have heard about the AMP trial from different sources, such as friends, relatives, and the project community team, these individuals did not impact their decision to enroll, but did provide avenues for additional information for decision-making.

During the AMP study, participants who were diagnosed with other health problems were treated more comprehensively at the research clinic by health workers. The longer they remained in the study, the more opportunities they had for medical attention. Likewise, the participants were reassured by management of any study-related health issues. This is viewed as a benefit because they saved time and received better service while in the study. This corresponds to findings from a study on gaps in universal health coverage in Malawi, which found that access to quality health services is a challenge to poor Malawians due to a shortage of medicine, health personnel, and geographical locations, which prompts them to not access proper assistance [19]. If potential participants rightly or wrongly believe that access to normal care, information, or treatment can be easily accessed through research participation, this highlights shortcomings with routine health services delivery and provision.

Additionally, the women expressed how they experienced an improved awareness of their individual sexual health, irrespective of not knowing whether they were given an active drug or a placebo. The study emphasized on prevention activities, including examining and treating women for sexually transmitted infections (STIs), and HIV testing and condom counselling. The HIV prevention education, HIV tests, and STI testing impacted their current relationships and promoted positive change behavior. Many talked about their commitment to using prevention strategies offered to avoid infection, apart from being in the study where they were receiving an investigational product (VRC01) for HIV prevention. This outcome is consistent with results from previous trials that reported participants´ engagement in lower HIV risky behaviors during trial follow-up beyond that observed in the source population [20-22]. Therefore, participation in the AMP clinical trial made the participants feel that they had more control over their health, which led to a more positive outlook and better quality of life.

According to the Malawi guidelines for research study participant remuneration, research remuneration is based on reimbursement of transport expenses and compensation for time and burden, but not as an incentive to participate in the study [23]. The AMP study participants received money equivalent to USD10 at each study visit as required by the Malawi National Health Sciences Research Committee (NHSRC) as fair compensation for transport and time [23]. Besides not having to pay for the drugs that are being investigated in the study or receiving free medical treatment for illnesses, some participants reported the stipend they were given in the study as a study benefit. This study provides further evidence that participants are motivated by monetary incentives such as the provision of a stipend. This also agrees with a study on motivational factors for participation in biomedical research, which was conducted in Blantyre, Malawi [13]. The transport reimbursement fund motivated their continued study participation and provided a means for sustaining their lives and families and improving their socioeconomic status. That is, time spent through study participation proved as valuable as other pursuits of income generation in a low-income setting. Therefore, most of the participants considered the clinical trial as a source of income since the majority of them were unemployed.

The perceptions and experiences of women taking part in HIV prevention trials were explored in Soweto, South Africa, where the central finding was that the participants felt a sense of empowerment in spite of their being embedded in a culture that has come to fear, deny, or ignore AIDS [11]. This was also reflected in this research, where participants volunteered to participate in the AMP study solely to assist society in developing scientifically validated approaches to end the epidemic. The participants indicated that by volunteering to take part in the study, they will assist Malawi in having evidence-based interventions to fight the virus, and they will feel proud to have been part of the drive.

The placebo-based intervention was a concern for some participants. Some participants felt knowing their treatment allocations would allow them to better protect themselves. However, as part of the study, they were encouraged to use the study-provided condoms, although there was an acknowledgement of inconsistent use of condoms. This gave them the surety that if not the study drug, at least their condom use would help them prevent HIV, although still, some reported inconsistent use of condoms because they believed they were being protected by the drug. Regardless, they felt it was better to be in the study and monitor their health, rather than not enrolling.

For some, the discomforts and uncertainty that came with participating in the study, like pain when the drip is being inserted, were outweighed by the benefit of being able to protect themselves long term. They were sure that even if something were to happen, for instance, side effects because of the study drug, they would be assisted by the clinic staff and did not see a need to stop taking part in the study.

Community stigma related to study participation came up from almost all those interviewed. They described that once people in the community learn of a person´s study participation, they are told negative things to discourage their continued participation. In the communities, people have different views of research. Some think of research as being a satanic activity, and they try to disassociate themselves with it, while others think it is a scheme by the researchers to steal blood from the participants through the samples that are collected. Community-level barriers such as mistrust of research and lack of perceived advantages to participation in a clinical trial were also cited by some studies that reported on the community´s role in influencing participants´ involvement in HIV prevention clinical trials [24,25]. However, regardless of what they were told, most participants still decided to continue taking part in the study because they had never experienced what they hear or heard of anyone in the study complain about the things the community described, and because they wanted to experience what was said for themselves and not just get influenced by other people.

Community educators and engagement were highlighted as critical to the research process. Community health workers in low, middle, and high-income countries, community health workers (CHWs) are a powerful force for promoting healthy behaviors and extending the reach of health systems around the world [26]. During the past decade, there has been an explosion of evidence concerning CHWs and their potential for improving population health where; health workforce resources are limited and access to basic services is low (mostly in low-income countries); and where large disparities in health outcomes exist between selected sub-populations and the population at large in spite of the presence of well-developed health systems (mostly in developed countries) [26]. Most participants in this study indicated that they knew about the AMP study through direct friends who are knowledgeable about health issues and are taking part in the AMP study or other studies at UNC, and the UNC Project community educators who were visiting the communities to sensitize people about the AMP study. The participants acknowledged that they could not have known about the AMP study had the CHWs not visited their areas. They also highlighted that CHWs hold the capacity to dispel community misconceptions and raise awareness regarding research.

**Study limitations:** the research study did not include participants who were not willing to join and those who dropped out of the AMP study. While this represented a small group, as the study had high retention, it was impossible to comprehend their motivations for leaving the study. Although the method used to collect data provided rich information from the perspective of participants, the results cannot be generalized. Furthermore, the use of purposive sampling to recruit participants was another limitation, although those interviewed were representative of the retained participants.

## Conclusion

The study aimed at understanding the reasons and experiences of women participating in the AMP study at a single research site in Lilongwe, Malawi. Thus, the study has provided insight into how to improve participation in the HIV clinical trials at UNC and other research institutions carrying out similar studies. This study has shown that participants in the AMP study enrolled and continued their participation because of perceived benefits such as access to quality health care and finances, and altruism. The participants´ enrolment in the study was facilitated by friends passing the information to each other, and the community educators who play an important role in participant recruitment and retention in HIV prevention clinical trials. When the participants´ study expectations are met, the chances of them adhering to the study visits and procedures are high. The UNC Project needs to continue providing other health services to the participants, as this is promoting participation in the trials.

### What is known about this topic

- Participation in HIV prevention clinical trials in sub-Saharan Africa is often motivated by access to healthcare services, financial compensation, and altruistic intentions to contribute to scientific advancement;
- Community perceptions, including misconceptions about research and concerns about blood sampling, can influence willingness to participate in clinical trials.

### What this study adds

- This study provides insight into the motivations and lived experiences of women participating in an antibody-mediated HIV prevention clinical trial in Malawi, highlighting the role of perceived health benefits, financial reimbursement, and altruism in sustaining participation;
- The findings emphasize the critical role of peer networks and community educators in facilitating recruitment and retention, while also documenting persistent community stigma and misconceptions about clinical research.
